# Mesenchyme-derived vertebrate lonesome kinase controls lung organogenesis by altering the matrisome

**DOI:** 10.1007/s00018-023-04735-6

**Published:** 2023-03-15

**Authors:** Salome M. Brütsch, Elizabeta Madzharova, Sophia Pantasis, Till Wüstemann, Selina Gurri, Heiko Steenbock, Amiq Gazdhar, Gisela Kuhn, Peter Angel, Saverio Bellusci, Jürgen Brinckmann, Ulrich auf dem Keller, Sabine Werner, Mattia R. Bordoli

**Affiliations:** 1 Department of Biology, Institute of Molecular Health Sciences, Swiss Federal Institute of Technology (ETH) Zurich, 8093 Zurich, Switzerland; 2 Department of Biotechnology and Biomedicine, Technical University of Denmark (DTU), 2800 Kongens Lyngby, Denmark; 3 Institute of Virology and Cell Biology, University of Lübeck, 23562 Lübeck, Germany; 4 Department of Pulmonary Medicine, University Hospital Bern, 3010 Bern, Switzerland; 5 Department of Health Sciences and Technology, Institute of Biomechanics, ETH Zurich, 8093 Zurich, Switzerland; 6 Division of Signal Transduction and Growth Control, DKFZ/ZMBH Alliance, German Cancer Research Center (DKFZ), 69120 Heidelberg, Germany; 7 German Lung Research Center (DCL), Giessen, Germany; 8 Department of Internal Medicine II, Universities of Giessen and Marburg Lung Center (UGMLC), Cardio-Pulmonary Institute (CPI), Aulweg 130, 35392 Giessen, Germany; 9 Department of Dermatology, University of Lübeck, 23562 Lübeck, Germany; 10 Department of Biomedical Research, University of Bern, 3010 Bern, Switzerland

**Keywords:** *Pkdcc*, Lung organogenesis, Collagen, Skull, Proteomics

## Abstract

Vertebrate lonesome kinase (VLK) is the only known secreted tyrosine kinase and responsible for the phosphorylation of a broad range of secretory pathway-resident and extracellular matrix proteins. However, its cell-type specific functions in vivo are still largely unknown. Therefore, we generated mice lacking the VLK gene (protein kinase domain containing, cytoplasmic (*Pkdcc*)) in mesenchymal cells. Most of the homozygous mice died shortly after birth, most likely as a consequence of their lung abnormalities and consequent respiratory failure. E18.5 embryonic lungs showed a reduction of alveolar type II cells, smaller bronchi, and an increased lung tissue density. Global mass spectrometry-based quantitative proteomics identified 97 proteins with significantly and at least 1.5-fold differential abundance between genotypes. Twenty-five of these had been assigned to the extracellular region and 15 to the mouse matrisome. Specifically, fibromodulin and matrilin-4, which are involved in extracellular matrix organization, were significantly more abundant in lungs from *Pkdcc* knockout embryos. These results support a role for mesenchyme-derived VLK in lung development through regulation of matrix dynamics and the resulting modulation of alveolar epithelial cell differentiation.

**Supplementary Information:**

The online version contains supplementary material available at 10.1007/s00018-023-04735-6.

## Introduction

The secreted tyrosine kinase VLK phosphorylates a large variety of secretory pathway-resident and extracellular proteins [1]. Mice harboring a global knockout of the VLK gene (*Pkdcc*) die shortly after birth, indicating an essential function for VLK in development [2, 3]. Their phenotype includes skeletal abnormalities triggered by delayed ossification, cleft palate, and lung hypoplasia, pointing to a key role for VLK in stromal cells [2, 3]. During bone development, VLK cooperates with Gli3, a component of the hedgehog (HH) signaling pathway, to control the kinetics of chondrocyte differentiation, and *Pkdcc* expression increased in the absence of HH signaling [4]. Another group reported that VLK negatively regulates HH signaling by promoting lysosomal degradation of smoothened, a key component of the HH signaling pathway [5]. VLK is highly abundant in α-granules of platelets, and upon its stimulated physiological release it phosphorylates co-released substrates [1]. Functionally, platelet-derived VLK plays a role in platelet aggregation as well as in the release of dense and α-granules, thereby promoting thrombus formation in mice upon arteriole damage [6]. In the nervous system, VLK-dependent phosphorylation of repulsive guidance molecule b (RGMb) drives axonal pathfinding and enables the accurate formation of neuronal circuitries [7]. Our recent studies demonstrated that hepatocyte-derived VLK is important for the prevention of perivascular fibrosis and inflammation in the liver, thereby revealing non-cell-autonomous activities of VLK in this tissue [8]. However, the specific functions of VLK in mesenchymal cells have not yet been determined. Therefore, we generated mice lacking *Pkdcc* in these cells. Because of the early postnatal lethality of the homozygous mutant mice, which most likely results from respiratory failure, we focused the analysis on late embryonic lung development.

Interestingly, development of epithelial structures, such as alveolar epithelial type II cell clusters and bronchi, was severely impaired, and the overall lung tissue appeared denser in *Pkdcc* knockout mice. Proteomics analysis revealed profound differences in extracellular matrix (ECM) proteome composition, suggesting that alterations in the mesenchyme-derived matrix affect lung epithelial development in a non-cell-autonomous manner.

## Materials and methods

### Mouse maintenance and mouse lines

Genetically modified mice were maintained under Specific Pathogen Free (SPF) conditions at the ETH Zurich Phenomics Center (EPIC). They were housed according to Swiss guidelines and received food and water ad libitum. All experiments with mice had been approved by the local veterinary authorities (Cantonal Veterinary Office Zurich). *Pkdcc*^fl/fl^ [9] females were bred with *Pkdcc*^fl/+^/*Col1a2-Cre*^±^ [10] males to obtain progeny containing *Pkdcc*^fl/fl^/*Col1a2-Cre*^−/−^, *Pkdcc*^fl/+^/*Col1a2-Cre*^−/−^, *Pkdcc*^fl/+^/*Col1a2-Cre*^±^ and *Pkdcc*^fl/fl^/*Col1a2-Cre*^±^ genotypes. The knockout mice were compared to mice carrying the *Pkdcc* floxed alleles, but lacking Cre recombinase. PDGFRa-H2B-eGFP mice were obtained from The Jackson Laboratory, Bar Harbor, ME. All mice were in C57BL/6 genetic background and of mixed sex. The exact sample size used in each experiment is indicated in the figure legends.

### Genotyping

Mouse genotyping was performed by polymerase chain reaction (PCR) analysis of genomic DNA, which had been isolated from tail biopsies obtained after sacrifice (embryos and neonates) or ear biopsies (adult mice) using the KAPA2G FAST Genotyping Mix (#KK5621, Roche, Rotkreuz, Switzerland). The following primers were used:

**Table:** 

Primer	Sequence forward primer	Sequence reverse primer
*mPkdcc*	CAC ACG CTC AAT CAT ACC ACA CC	GGT CAT TAG GTC ACA GGG TAG GG
*mCol1a2-Cre*	TTA GCA CCA CGG CAG CAG GAG GTT	CAG GCC AGA TCT CCT GTG CAG CAT
*mPDGFRa-eGFP*	CCC TTG TGG TCA TGC CAA AC	GCT TTT GCC TCC ATT ACA CTG G ACG AAG TTA TTA GGT CCC TCG AC

### Protein extraction from lung tissue for proteomics analysis

Lung tissue samples from mice at E18.5 were soaked in 4 M guanidine chloride, 250 mM HEPES pH 7.8, supplemented with 1 × PMSF. Samples were processed using pressure cycling technology (PCT) as previously described [11], sonicated, (3 × 10 cycles; 30 s ON, 30 s OFF) at 4 °C, and centrifuged for 10 min at 10,000 × g and 4 °C. The supernatants containing the extracted proteins were transferred to a new Eppendorf tube, and the buffer was adjusted to 2.5 M guanidinium chloride, 250 mM HEPES pH 7.8. Samples were stored at – 80 °C before proceeding according to the quantitative proteomics workflow.

### Quantitative proteomics

For 8plex-TMT quantitative proteomics analysis, we applied protein-level labeling, following a previously described workflow [12]. Samples were analyzed after trypsin digest. The protein samples (50 μg per condition) were first denatured by incubation for 15 min at 65^o^ C. Cysteine residues were reduced by adding 3.5 mM Tris (2-carboxyethyl) phosphine (TCEP) and incubation for 45 min at 65 ^o^C, and then alkylated by adding 5 mM of chloroacetamide (CAA) for 30 min at 65 ^o^C. The proteins in each sample were labeled at a 1:4 protein: TMT (w/w) ratio with TMT reagents (TMT10plex labeling Kit; Thermo Fisher Scientific, Waltham, MA) for 1.5 h at room temperature (RT), after which the labeling reactions were quenched with 100 mM NH_4_HCO_3_ for 30 min. The labeled samples were then pooled and precipitated by adding seven sample volumes of ice-cold acetone and one sample volume of ice-cold methanol and incubated for 2 h at – 80 ^o^C. The samples were centrifuged at 4700 × g at 4 ^o^C for 30 min, washed with 5 ml ice-cold methanol, and centrifuged again. The pellet was air-dried, resuspended in 100 mM NaOH, and adjusted with 1 M HEPES, pH 7.8 to 1 mg/ml protein in 100 mM HEPES, pH 7.8. The protein samples were digested with trypsin (Trypsin Gold, V5280, Promega, Madison, WI; 1:100 enzyme: protein ration (w/w)) for 16 h at 37 ^o^C.

### Desalting of unfractionated peptides

Before peptide fractionation, peptide samples were desalted with Sep-Pak C18 columns (Waters Corporation, Milford, MA). Columns were activated with 0.9 ml of 100% methanol, cleaned with 0.9 ml of 80% acetonitrile (ACN), 0.1% formic acid (FA), and equilibrated with 3 × 0.9 ml of 3% ACN, 1% trifluoroacetic acid (TFA). Next, the samples were acidified with 1% TFA and loaded on the column. The columns were washed with 3 × 0.9 ml 0.1% FA, after which the samples were eluted with 3 × 200 μl 80% ACN, 0.1% FA. The eluted peptides were dried under vacuum and stored at – 20 ^o^C.

### Peptide fractionation and LC–MS/MS

Peptide mixtures were fractionated using a Dionex UltiMate 3000 UHPLC (Thermo Fisher Scientific) coupled to an Acclaim^™^ PA2 nano HPLC column (3 μm, 150 × 0.3 mm, Thermo Fisher Scientific). Samples were resuspended in 5 mM NH_4_HCO_3_, pH 10, and fractionated with the following gradient: 2 min 5% B; 50 min 35% B; 58 min 70% B; 65 min 70% B; 70 min 5% B with eluent A (5 mM NH_4_HCO_3_) and eluent B (100% ACN) at a flow rate of 5 μl/min. Forty-five fractions were collected using a Dionex AFC-3000 fraction collector in a 96 deep-well plate and subsequently pooled into 22 samples. The peptide fractions were analyzed on a Q Exactive HF-X mass spectrometer coupled to an LC Evosep One system. They were loaded onto Evotips (Evosep, Odense, Denmark), according to the manufacturer’s instructions. Briefly, the Evotips were washed with Solvent B (80% ACN, 0.1% FA) and centrifuged for 1 min at 700 × g. Next, the tips were soaked for ~ 1 min in 1-propanol, equilibrated with Solvent A (0.1% FA), and centrifuged for 1 min at 700 × g. The samples were loaded and centrifuged for 1 min at 700 × g. Subsequently, they were washed with Solvent A and centrifuged. Finally, 100 μl Solvent A were added to the Evotips to prevent them from drying before injecting into the mass spectrometer. After the samples were loaded, they were analyzed with a pre-programmed 44 min gradient per injection using an Acclaim^™^ PepMap^™^ RSLC C18 column (2 μm, 75 μm × 150 mm, Thermo Fisher Scientific) at RT. Data was recorded in data-dependent acquisition (DDA) mode. A precursor MS1 scan (m/z 350–2000) was acquired at a resolution of 120,000 with an AGC target 3e6 and a maximum fill time of 50 ms. The 20 most abundant precursor ions were selected from each MS scan for a subsequent higher-energy collision-induced dissociation (HCD) fragmentation with a normalized collision energy (NCE) of 30%. Fragmentation was performed at resolution 45,000 with an AGC target of 1e5 and an injection time of 96 ms, using a precursor isolation window of 0.7 m/z and a dynamic exclusion of 20 s after single isolation and fragmentation of a given precursor.

### Data analysis and normalization

Raw files were searched by Sequest HT from Proteome Discoverer 3.0 (Thermo Fisher Scientific) against the mouse UniProt database (sp_canonical TaxID = 10090, v2022-01-30; 17067 sequences). The following parameters were used for database searches: semi-ArgC for enzyme specificity, allowing one missed cleavage; carbamidomethyl (C) and TMT6plex (K) as fixed modifications, and acetyl (N-term), TMT6plex (N-term), pyroQ (N-term), deamidation (NQ), oxidation (MP), and phosphorylation (Y) were set as variable modifications: precursor mass error tolerance of 10 ppm and fragment mass error at 0.02 Da. Percolator was used for decoy control and FDR estimation (0.01 high confidence peptides, 0.05 medium confidence). TMT6plex-modified N-terminal modified and tyrosine phosphorylated peptides were excluded from protein quantification, data were normalized to ‘Total Peptide Amount’, scaling performed with mode ‘On All Average’, and differential abundance determined with ‘Protein Abundance Based’ ‘Protein Ratio Calculation’ and applying Proteome Discoverer’s ‘ANOVA (Individual Proteins)’ setting.

### Hydroxyproline analysis

Collagen content of the lung was analyzed as described previously [12, 13]. Harvested lungs were weighed, snap frozen and then homogenized in phosphate buffered saline (PBS). 1 ml of the homogenate was treated with 10% trichloric acid (TCA), then hydrolyzed with 6 M hydrochloric acid for 18 h at 110 ^o^C, and the pH was adjusted to 7. The oxidation process was started by 20 min incubation with 1 ml of chloramine T reagent at RT and stopped by addition of 1 ml of 3.15 M perchloric acid. Samples were then incubated in Ehrlich reagent (p-dimethylaminobenzaldehyde added to methyle cellusolve) for 20 min at 55–65 ^o^C. Finally, the absorbance of each sample was measured at 557 nm, and a standard curve was calculated using known concentrations of reagent grade hydroxyproline (Sigma) as described before.

### Collagen cross-link and protein analysis

Analysis of collagen and of collagen cross-links was performed as reported previously [15]. Briefly, both lungs from two animals were pooled and treated with sodium borohydride (Sigma, 25 mg NaBH_4_/ml in 0.05 M NaH_2_PO_4_/0.15 M NaCl pH 7.4, 1 h on ice, 1.5 h at RT) to stabilize reducible acid-labile cross-links, digested for 12 h at 37 ^o^C with high purity bacterial collagenase (C0773; Sigma, 50 U/ml) and hydrolyzed in 6 N HCl at 110 °C for 24 h. The hydrolysates were precleared by solid phase extraction and analyzed on an amino acid analyzer (Biochrome30, Biochrome, Cambridge, UK). Quantification was based on ninhydrin-generated leucine equivalence factors (DHLNL, HLNL: 1.8). The nomenclature used in the manuscript refers to the reduced variants of cross-links (DHLNL, HLNL). For protein analysis, specimens were digested with bacterial collagenase. After centrifugation, the soluble fraction containing collagen was subjected to hydrolysis and amino acid analysis. Collagen content was calculated based on a content of 14 mg hydroxyproline in 100 mg collagen. The residual fraction was extracted with hot alkali (0.1 N NaOH, 95 °C, 45 min). After centrifugation, the supernatant containing non-collagen/non-elastin proteins and the insoluble residue containing elastin were subjected to hydrolysis and amino acid analysis.

### Tissue and cell processing

Tissue samples were fixed overnight at 4 °C in acetic ethanol (25% acetic acid glacial, 75% ethanol) or 4% paraformaldehyde (PFA) (#P6148, Sigma), embedded in paraffin, and sectioned (3.5 µm thickness). Alternatively, fresh tissue was immediately frozen in tissue freezing medium® (#14020108926, Leica Biosystems, Wetzlar, Germany) and sectioned (5 µm thickness).

### Immunofluorescence staining

Sections from lung tissue, which had been fixed with PFA or acetic ethanol, were dewaxed using xylene and rehydrated using an ethanol gradient. PFA sections were subjected to an antigen retrieval step performed by incubation in citrate buffer (10 mmol/l citric acid pH 6) at 95 °C for 1 h, followed by three washes with PBS containing Tween (PBS-T; 137 mM NaCl, 2.7 mM KCl, 10 mM Na_2_HPO_4_, 2 mM KH_2_HPO_4_; 0.1% Tween). All samples were blocked with 10% bovine serum albumin (BSA) (#P06-1391100, PAN Biotech, Aidenbach, Germany) in PBS for 30 min at RT, followed by incubation with the primary antibody overnight at 4 °C and for 2 h with the secondary antibody at RT. Hoechst 33342 (1:1000 diluted) was used to counterstain nuclei. The samples were mounted with Mowiol-DABCO.

The following antibodies were used for immunostaining: rabbit anti-VLK 404 (Whitman laboratory, Harvard University, Boston, MA), goat anti-cytokeratin 19 (Hybridoma Product TROMA-III; Developmental Studies Hybridoma Bank (DSHB), Iowa City, IA), rabbit anti-SPC (#sc-13979, Santa Cruz, Santa Cruz, CA), rat anti-Ki-67-FITC (#11-5698-82, Thermo Fisher Scientific), rabbit anti-fibromodulin (#ab81443, Abcam, Cambridge, UK), mouse-anti podoplanin (#8.1.1.; DSHB), rabbit-anti-SOX9 (#AB5535 Millipore, Darmstadt, Germany), rat anti-CD31-phycoerythrin (PE) (#553370, BD Pharmingen, San Diego, CA), rabbit anti-matrilin-4 (#ab106379, Abcam), rabbit anti-cleaved caspase 3 (#9661, Cell Signaling, Danvers, MA), guinea pig anti-pan keratin (GP14, Progen Biotechnik GmbH, Heidelberg, Germany), goat anti-PDGFR alpha (AF1062, R&D Systems, Minneapolis, MN), donkey anti-rabbit-Cy3 (#711-165-152, Jackson ImmunoResearch, West Grove, PA), donkey anti-rat-Cy3 (#712-165-150, Jackson ImmunoResearch), bovine anti-goat-Cy3 (#805-165-180, Jackson ImmunoResearch), goat anti-guinea pig-Cy2 (#106-225-003, Jackson ImmunoResearch) and donkey anti-goat-Cy2 (#705-225-147, Jackson ImmunoResearch).

### Haematoxylin and eosin (H&E), Sirius red and Herovici staining

Acetic ethanol- or PFA-fixed paraffin sections were deparaffinized, rehydrated using a xylene/ethanol gradient, stained with haematoxylin (#3870, JT Baker^®^, Phillipsburg, NJ) and eosin-Y alcoholic (#102439, Merck, Darmstadt, Germany), Sirius Red (Direct Red 80, #365548, Sigma), or using the Herovici procedure [16], and mounted with Eukitt^®^ (#03989, Sigma).

### Quantification of Sirius red und Herovici staining

ECM patterns based on Sirius Red staining were analyzed in Fiji v1.53t using TWOMBLI (version April 2022) [17]. Three representative images were used as a test set to determine optimal parameters: contrast saturation (0.35), line width (5), curvature window (40), minimum branch length (15), and maximum display HDM (200). Gap analysis was excluded.

Herovici-stained areas were quantified using QuPath Version 0.40 [18]. A pixel classifier based on an artificial neural network (ANN_MLP) was trained on a representative subset of the analyzed images to identify areas of young collagen. Young collagen was calculated in relation to total stained tissue area.

### Isolation and culture of primary mouse embryonic fibroblasts (MEFs) or lung fibroblasts

MEFs or primary lung fibroblasts were isolated from E18.5 embryos. Embryos were decapitated. For MEF isolation, the head, liver and heart were removed, and the remaining tissue was minced with a razor blade. For lung fibroblasts, the lung was isolated and also minced with a razor blade. Minced tissue was incubated with 2 × trypsin (#59418C, Sigma) for 15 min in a water bath at 37 °C. The suspension was then centrifuged, and afterwards the supernatant was discarded. Cells were then cultured in 6-well plates in DMEM (#6429, Sigma) containing 10% fetal bovine serum (FBS, #A4766801 Thermo Fisher Scientific) and 1% penicillin–streptomycin (Sigma) at 37 °C, 5% CO_2_.

### RNA isolation and RT-qPCR analysis

RNA was isolated using TRIzol (#15596026, Thermo Fisher Scientific) using the manufacturer’s protocol. cDNA was synthesized using iScript (#1708891, BioRad, Hercules, CA). RT-qPCR was performed using the LightCycler®480 SYBR Green I Master reaction mix (Roche), and data (Ct-values) were collected using the LightCycler®480 software. All samples were measured in duplicates and gene expression was determined using the 2^−ΔΔCt^ method. Data were normalized to the expression levels of the gene encoding ribosomal protein 29 (*Rps29*).

**Table:** 

Primers	Sequence forward primer	Sequence reverse primer
*Pkdcc*	CAA GCT GCT CAA AGA GAT GGT	TGG TAG CAA TAG CCA TAG AGC TG
*Fmod*	CAG GGC AAC AGG ATC AAT G	CTG CAG CTT GGA GAA GTT CAT
*Matn4*	GGC GAT CCA GTA CGC TAT GAA	GGC CAA ACT CCT GGA TGA GA
*Timp1*	GCC CCC TTT GCA TCT CTG GCA T	TGC GGC ATT TCC CAC AGC CT
*Col1a1*	TGT TCA GCT TTG ACC TCCC GGCC T	TCT CCCC TTG GGT CCC TCG ACT
*Col3a1*	TCC CCT GGA ATC TGT GAA TC	TGA GTC GAA TTG GGG AGA AT
*Hrg*	CAC CAA CTG TGA TGC TTC TGA	AGT AGT AGA CTG TGG CCG TTC C
*C4b*	CCT GGG TGT TCA GCT TCT GT	CAG GAA CCA CCC TTT GGG TT
*Cfh*	TTA CCG TGA ATG TGG TGC AGA	GCT CCA AAG GCC ATT TTC TGA
*Fgg*	GGT CAC CCA GAC ACC ATG AG	GGT TGG GCA GAA ACT ACC GA
*Rbp4*	ACA AGG CTC GTT TCT CTG GG	TGT GAA AGT GCC CAC CAT GT
*Fth1*	ACC TGG AGT TGT ATG CCT CCT	AGG AAG ATT CGG CCA CCT
*Rps29*	GGT CAC CAG CTC TAC TG	GTC CAA CTT AAT GAA GCC TAT GTC C
*Ccnd1*	ACT GCC GAG AAG TTG TGC AT	AAG CAG TTC CAT TTG CAG CAG
*Smo*	GCA AGC TCG TGC TCT GGT	TCC ACT CGG TCA TTC TCA CA
*Ptch1*	TGG AGC AGA TTT CCA AGG GGA	GCC CCA AAT ATG AGG AGA CCC
*Gli1*	GTA TGA GAC AGA CTG CCG CT	GCT CAC TGT TGA TGT GGT GC
*Azgp1*	TCA CCC CAG ACA TCA ACT CCT A	GGT CTA AGG GGA TCC AAG CTG
*Pdpn*	GGA GGG CTT AAT GAA TCT ACT G	GTT GTA CTC TCG TGT TCT CTG
*Scgb1a1*	AAG ATC GCC ATC ACA ATC AC	CTT CAG GGA TGC CAC ATA AC
*Fgb*	AAG CTG CCG ATG ATG ACT ACG	CGA TAG CCC CCT CCA CTG ATA
*Plod1*	CCA CAA AAG AGA CTG AGG GC	CAT CCA CAC TCC AGT CCT CC
*Plod2*	TGA TGG ATT CCA CAG ATT TAT GA	CCC CTC CGA TAC TGT TCA TT
*Plod3*	ATT GCT GGT GAT CAC TGT GG	TCC TTC TTG AGC CAC CTG AC

### Image acquisition and quantification

Fluorescence stainings were imaged using an Axioskop 2 fluorescence microscope (Carl Zeiss, Inc., Oberkochen, Germany), and the corresponding software (Carl Zeiss, Inc.). Image acquisition was performed with an Axiocam HRc camera (Carl Zeiss, Inc.) connected to the microscope. Histochemical stainings were imaged with a Pannoramic 250 slide scanner (3DHISTECH, Budapest, Hungary). Quantifications were performed using the Fiji software [19].

### Computer tomograph (CT) scan

The heads from E18.5 CTRL and *Pkdcc*^−/−^ embryos were fixed in 4% PFA for 24 h and then washed and stored in PBS. They were scanned on a vivaCT80 (Scanco Medical AG, Brüttisellen, Switzerland). 1000 projection images were obtained at an energy of 45 kVp, a current of 177 µA and an integration time of 350 ms. Density calibration of the scanner is checked weekly. Images were reconstructed at an isotropic voxel size of 10.4 µm. After filtration (Gauss filter, sigma 1.2, support 1), a threshold of 150 mgHA/cm^3^ was applied to segment bone from soft tissue. Because of the low threshold, noise in the soft tissue remained, which was removed partially by a component labelling that excluded components containing less than 20 voxels. Images were visualized in 3D using the software of the scanner (µCT Ray, Scanco Medical AG).

### Statistical analysis

Statistical analysis was performed using Prism 8 software (GraphPad Inc., San Diego, CA). Differences between groups were analyzed using Mann–Whitney test (n ≥ 4 or unpaired t test (n = 3)).

## Results

### VLK is expressed in the developing lung

**Fig. 1:**
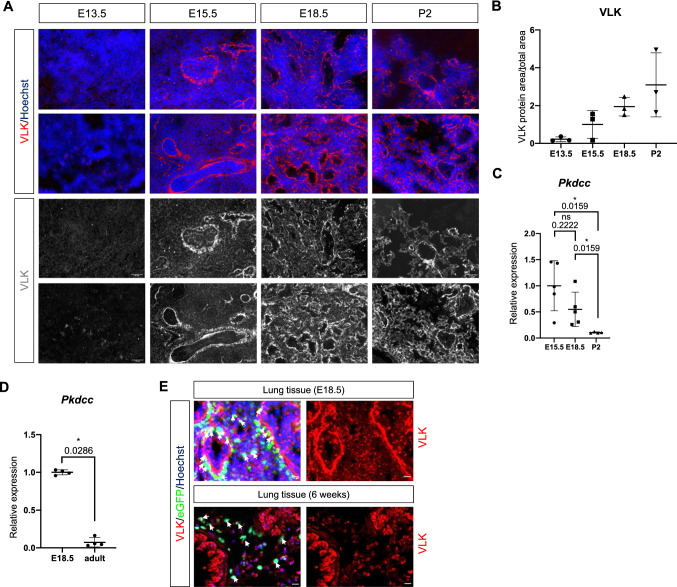
VLK is highly expressed in the lung during late embryonic development. **A**, **B** Representative VLK immunofluorescence stainings of sections from the lung of wild-type mice at different stages of embryonic development (red or white) and counterstaining of nuclei with Hoechst (blue). Scale bars: 50 μm. **C**, **D** RT-qPCR analysis of RNA samples from total lung tissue of wild-type mice at different stages of lung development (embryonic day (**E**) 13.5–18.5; **C**, **D**) and of postnatal (P2, **C**) and adult mice (**D**) for *Pkdcc* relative to *Rps29*. N = 4–5 mice per time point. **E** Representative VLK immunofluorescence stainings (red) of lung sections from E18.5 or adult PDGFRa-eGFP mice. eGFP-positive mesenchymal cells are labelled in green. eGFP/VLK double positive cells are indicated with white arrows. Nuclei were counterstained with Hoechst (blue). Scale bars: 20 μm (E18.5) and 50 μm (adult). Bar graphs show mean ± S.D. P values are indicated in the graphs; statistical analysis was performed using Mann–Whitney U test

Based on the previously reported *Pkdcc* expression in mesenchymal and mesothelial cells of the lung and the abnormalities in lung development that were observed in mice with global *Pkdcc* knockout [3], we studied the expression and function of VLK in this tissue. Consistent with published RNA data [3], immunofluorescence staining using an antibody with previously confirmed specificity for VLK [8] showed expression of VLK in the developing lung. Only weak staining was observed at embryonic day 13.5 (E13.5), but the staining intensity continuously increased until postnatal day 2 (P2) (Fig. 1A, B and Supplementary Fig. 1A for secondary antibody staining). mRNA quantification using whole lung tissue revealed a decrease in *Pkdcc* expression after birth (Fig. 1C), and a further decline in adult lung (Fig. 1D). These results demonstrate that VLK expression peaks around birth and suggest a role for VLK during the canalicular (E16.5-E17.4) and saccular stages (E17.5-P5) of lung development, when the respiratory tree is further expanded, the terminal bronchioles are divided into respiratory bronchioles and alveolar ducts, and the interstitium is thinned as a consequence of apoptosis and differentiation of mesenchymal cells [17, 18].

Immunofluorescence staining of lung sections from mice that express nuclear enhanced green fluorescent protein (eGFP) in mesenchymal cells of all organs under the control of the platelet-derived growth factor receptor alpha (*Pdgfra*) promoter identified VLK in eGFP-positive cells of E18.5 lungs, confirming VLK expression in cells of mesenchymal origin (Fig. 1E). However, VLK staining was also detected in other cells of the developing lung, in particular in epithelial cells. No signals were obtained when sections were only stained with the secondary antibody (Supplementary Fig. 1A). In adult lung tissue, VLK staining was still observed in epithelial cells, but the number of eGFP-positive mesenchymal cells as well as the VLK staining intensity in the mesenchyme had decreased (Fig. 1E; Supplementary Fig. 1B for secondary antibody staining only). Together with the RNA data, this finding demonstrates that VLK expression declines in the lung after birth, in particular in mesenchymal cells, and suggests that VLK in mesenchymal cells is most important during late lung organogenesis.

### Deletion of *Pkdcc* in mesenchymal cells results in smaller body size, craniofacial abnormalities and early postnatal lethality

**Fig. 2:**
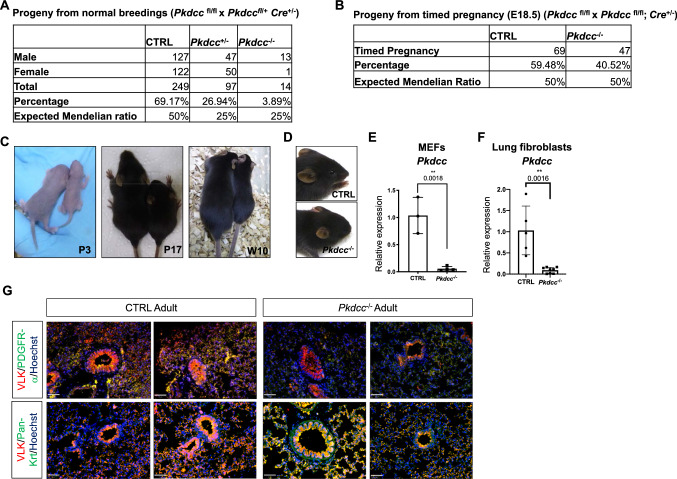
*Pkdcc* deletion in mesenchymal cells leads to smaller body size, craniofacial abnormalities and high postnatal lethality. **A**, **B** Expected and observed ratios between genotypes in the progeny of *Pkdcc*^fl/fl^ x *Pkdcc*^fl/+^; *Col1a2-Cre*^±^ mice after weaning (**A**) or in the progeny of *Pkdcc*^fl/fl^ x *Pkdcc*^fl/fl^; *Col1a2-Cre*^±^ mice at E18.5 (**B**). **C**, **D** Whole-body images (**C**) and head close-up (**D**) of CTRL (left) and *Pkdcc*^−/−^ (right) mice taken at the indicated ages. **E**, **F** RT-qPCR analysis of RNA samples from primary mouse embryonic fibroblasts (MEFs; **E**) and primary lung fibroblasts (**F**) from E18.5 CTRL and *Pkdcc*^−/−^ mice for *Pkdcc* relative to *Rps29*. Bar graphs show mean ± S.D. P values are indicated in the graphs; statistical analysis was performed using unpaired t test. N = 3–4 MEF, 5–8 lung fibroblasts; each culture was from a different embryo. **G** Representative co-immunofluorescence stainings for VLK (red), PDGFR alpha (green) or pan-keratin (Krt) (green) on lung sections from adult CTRL and *Pkdcc*^−/−^ mice. Double positive cells appear yellow. Nuclei were counterstained with Hoechst (blue). Scale bars: 50 μm

To investigate the role of mesenchyme-derived VLK in lung organogenesis, we crossed mice with floxed *Pkdcc* alleles [9] with transgenic mice that express *Cre* under the control of the *Col1a2* promoter [10]. We and others previously showed that this mouse line allows specific deletion of floxed alleles in cells of mesenchymal origin when male *Col1a2*-Cre mice are used [10, 19]. Surprisingly, the *Col1a2*-Cre-driven *Pkdcc* knockout (*Pkdcc*^−/−^) caused lethality of most of the mice. Only very few homozygous knockout mice survived the first night or even reached adulthood, with the majority being males (Fig. 2A). Chi-square test revealed a significant difference between the observed and expected Mendelian ratio (Supplementary Fig. 1C). However, when we performed timed pregnancies and analyzed the embryos at E18.5, the knockout mice were present in the expected Mendelian ratio (Fig. 2B). E18.5 knockout embryos included mice of both sexes, although seven out of ten knockout embryos that were tested for their sex were also males. These findings suggest that *Pkdcc*^−/−^ mice die shortly after birth, possibly from respiratory failure. The few surviving homozygous *Col1a2-Pkdcc*^*−/−*^ mice were smaller than control mice (mice with floxed *Pkdcc* alleles, but without *Cre* allele) or heterozygous littermates, both at birth and during adulthood (Fig. 2C). Moreover, they presented craniofacial abnormalities, resulting in a ‘rounded’ head (Fig. 2D). Heterozygous littermates did not show an obvious macroscopic phenotype. This new mouse line therefore has some similarities with the previously reported global *Pkdcc* knockout line obtained with transgenic mice in which *Cre* expression is under the control of the ubiquitously active *Ella* promoter [3], suggesting that major phenotypic traits of the global knockout mice result from the loss of VLK in mesenchymal cells. The efficient *Pkdcc* knockout was confirmed in MEFs (Fig. 2E) and in lung fibroblasts (Fig. 2F) from E18.5 embryos. Co-immunofluorescence staining with markers for epithelial cells (pan-keratin) or mesenchymal cells (PDGFRα) showed that VLK was almost undetectable in mesenchymal cells of adult *Pkdcc*^*−/−*^ mice, while epithelial cells were still VLK positive (Fig. 2G, Supplementary Fig. 1D). These findings suggest the expected mesenchyme-specific deletion of *Pkdcc*, although the signal intensity in some bronchi was also reduced. This could be a consequence of the loss of mesenchyme-derived VLK that also reaches epithelial cells, but some deletion in epithelial cells cannot be fully excluded.

Following the initial characterization of the knockout mice and considering the high lethality, breedings leading to *Col1a2-Pkdcc*^*−/−*^ mice in the litters were stopped for animal welfare reasons, in line with 3R regulations. We therefore performed timed matings and focused on E18.5 embryos, because this time point represents the peak of embryonic VLK expression (Fig. 2A, B).

### Loss of mesenchyme-derived VLK causes skeletal and lung abnormalities

Based on the previously demonstrated skeletal abnormalities of the mice with global *Pkdcc* knockout, we performed computed tomography (CT) scans of the heads from E18.5 embryos. The images confirmed the rounded shape of the head in the *Pkdcc*^−/−^ mice. The top of the calvaria appeared rounded compared to CTRL mice, while the facial bones were shortened, resulting in brachycephaly (Supplementary Fig. 2A, B). Furthermore, the distance between left and right palate bone was increased. No increase in the distance was observed between the rostral bones, which form the palate, the palatine processes of the maxillae and the *ossa incisive.* These features may indicate cleft palate, which would, however, only be present in the caudal part (Supplementary Fig. 2C). Overall, the phenotype of the skull appears milder than in the global *Pkdcc* knockout pups published by Kinoshita et al. [3], which is consistent with a cell type-specific deletion.

**Fig. 3:**
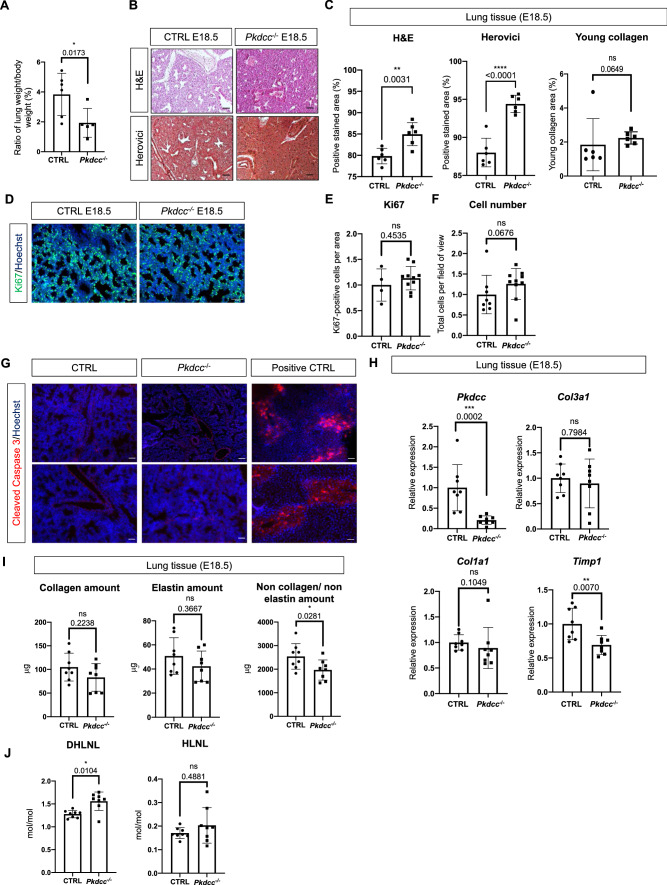
Loss of mesenchyme-derived VLK causes severe lung abnormalities. **A** Ratio of lung weight of E18.5 embryos relative to whole body weight. N = 4–5. Each image depicts a single mouse. **B**, **C** Representative photomicrographs (**B**; Scale bars: 50 mm) and quantification of positively stained area relative to total lung area in lung sections from E18.5 CTRL and *Pkdcc*^−/−^ mice using the indicated histological stainings or measuring young collagen based on Herovici stainings (**B**). N = 6 mice per genotype. **D–F** Representative immunofluorescence stainings of mouse lung sections from E18.5 CTRL and *Pkdcc*^*−*/−^ mice for Ki67 (**D**, Scale bars: 50 μm) and quantification of Ki67-positive cells per area (**E**) or total nuclei count per field of view (**F**). N = 4–10 mice per genotype. **G** Representative immunofluorescence stainings of mouse lung sections from E18.5 CTRL and *Pkdcc*^*−*/−^ mice for cleaved caspase 3. Liver sections from mice treated with the hepatotoxin CCl_4_ were used as a positive control (Scale bars: 100 μm). **H** RT-qPCR analysis of RNA samples from E18.5 lung tissue of CTRL and *Pkdcc*^−/−^ mice for *Pkdcc*, *Col3a1*, *Col1a1* and *Timp1* relative to *Rps29*. N = 8 per genotype. **I, J** Biochemical analysis of matrix proteins (**I**) or collagen cross-links (**J**) in E18.5 lungs. N = 8 per genotype, for each data point two embryos were pooled. Bar graphs indicate mean ± S.D. P values are indicated in the graphs; statistical analysis was performed using Mann–Whitney U test. Each image depicts a single mouse

Analysis of the lung showed a reduced ratio of lung to body weight in the mutant mice (Fig. 3A). Histologically, hematoxylin/eosin and Herovici stainings of lung sections revealed an altered and denser lung structure in *Col1a2-Pkdcc*^*−/−*^ homozygous embryos at E18.5 compared to control littermates (Fig. 3B, C), although the Herovici staining did not reveal differences in young collagen (Fig. 3C). The enhanced tissue density was also not a consequence of enhanced cell proliferation at this time point. Neither the number of Ki67-positive cells nor the total cell number (determined based on the number of nuclei) in whole lung tissue differed between genotypes (Fig. 3D–F). Cleaved caspase 3-positive (apoptotic) cells were not detectable in CTRL or *Pkdcc*^*−/−*^ homozygous embryos at E18.5 (Fig. 3G). These data suggest that the denser lung structure results from alterations in the deposited ECM. RT-qPCR analysis of RNA from total E18.5 lung tissue for markers of collagen-dominated fibrosis, such as collagen 1α1 (*Col1a1*) or collagen 3α1 (*Col3a1*), did not reveal differences between genotypes. There was even a significant decrease in the expression of the fibrosis marker TIMP metallopeptidase inhibitor 1 (*Timp1)* in lung tissue of *Pkdcc*^−/−^ mice (Fig. 3H). Together, this data does not support a classical fibrotic phenotype [20, 21]. Expression of these genes was also not significantly altered in cultured primary MEFs from *Pkdcc*^−/−^ mice (Supplementary Fig. 3A). In line with the mRNA data, collagen staining of E18.5 lung sections using Sirius Red did not show obvious differences in staining intensity, area of collagenous ECM, collagen branch points, high density matrix or collagen fiber alignment between genotypes (Supplementary Fig. 3B, C). The similar amounts of collagen and elastin in E18.5 lung tissue were confirmed by biochemical quantification. The total amount of non-collagen and non-elastin proteins was even slightly, but significantly reduced in lung tissue from *Pkdcc*^−/−^ mice (Fig. 3I), suggesting that alterations in the composition and/or organization of the ECM rather than a global increase in ECM proteins are responsible for the phenotypic abnormalities. Biochemical analysis of E18.5 lung tissue further revealed an increase in the hydroxylysine-derived collagen cross-link dihydroxylysinonorleucine (DHLNL), demonstrating that the deposited collagen is differently cross-linked. This may have functional consequences, because an increase in DHLNL is associated with a stiffer ECM [25]. Hydroxylysinonorleucine (HLNL) collagen cross-links were not affected in *Pkdcc*^−/−^ lung tissue (Fig. 3J). To determine if the increase in DHLNL cross-links is a consequence of increased expression of genes encoding the lysine hydroxylases procollagen-lysine,2-oxoglutarate 5-dioxygenases 1–3 (*Plod1*, *Plod2*, *Plod3*), we analyzed their expression in cultured primary E18.5 lung fibroblasts, but we did not detect a significant difference between genotypes. In vivo, *Plod1* and *Plod3* expression was even reduced in total lung tissue. Only expression of *Plod2* was increased, but the variability in the knockout mice was rather high (Supplementary Fig. 3D, E). Overall, these results suggest that changes in the expression of PLODs are not responsible for the increased DHLNL levels.

Histological analysis of lung tissue from adult heterozygous mice did not show an obvious phenotype (Supplementary Fig. 4A, B), and neither expression of *Col1a1*, *Col3a*1 and *Timp1* nor the total levels of hydroxyproline were affected (Supplementary Fig. 4C, D).

### Loss of VLK in mesenchymal cells affects differentiation of lung epithelial cells

**Fig. 4:**
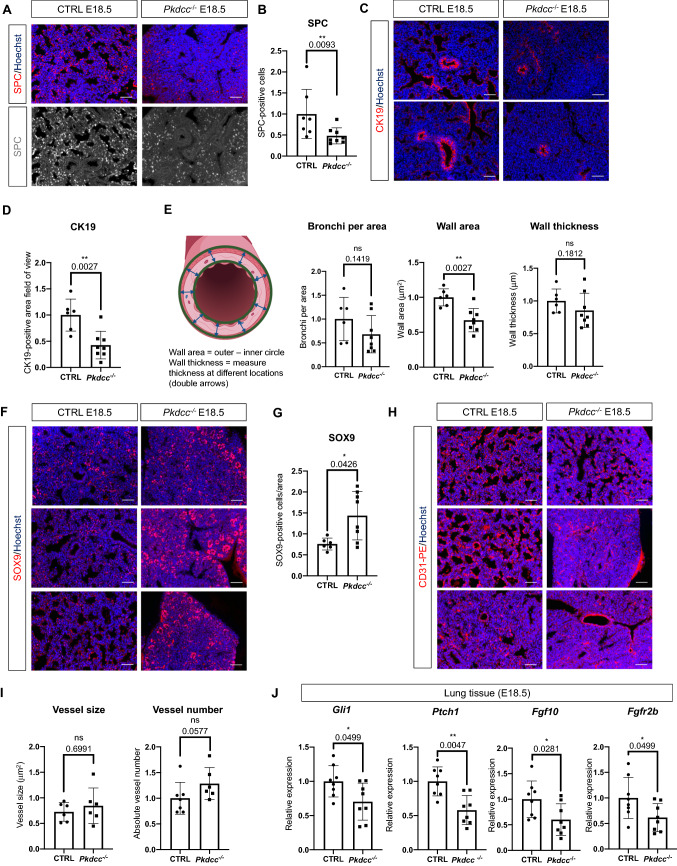
*Pkdcc* deletion in mesenchymal cells severely affects epithelial cells in the developing lung. **A-I** Representative immunofluorescence stainings of E18.5 lung sections from CTRL and *Pkdcc*^−/−^ mice for SPC (**A**), CK19 (**C**), SOX9 (**F**) or CD31 (**H**) (red), counterstained with Hoechst (blue), and quantification of SPC-positive cells (**B**; N = 7–8 mice per genotype), total CK19-positive area (**D**), number of bronchi per area, CK19-positive wall area and wall thickness (**E**; N = 6–8 mice per genotype), SOX9-positive cells (**G**; N = 6–8 mice per genotype); or size and number of CD31-positive vessels (**I**; N = 6–7 mice per genotype). The cartoon in **E** indicates the parameters that were measured to obtain the wall area and wall thickness. Created with BioRender. **J** RT-qPCR analysis of RNA samples from lung tissue of E18.5 CTRL and *Pkdcc*^−/−^ mice for *Gli1*, *Ptch1*, *Fgf10* and *Fgfr2b* relative to *Rps29*. N = 8 per genotype. Scale bars: 50 μm. 2–5 images per mouse were analyzed. Bar graphs show mean ± S.D. P values are indicated in the graphs; statistical significance was determined using Mann–Whitney U test

Next, we investigated whether loss of VLK in mesenchymal cells affects epithelial cell differentiation. This is relevant, since during the saccular stage alveolar epithelial cells (AEC) differentiate into alveolar type I (AECI; pneumocytes) and type II epithelial cells (AECII) [26]. The latter secrete surfactant, thereby reducing tension within the lung upon inhalation [27]. Lung tissue from E18.5 *Pkdcc*^−/−^ embryos showed significantly less cells that were positive for the AEC II cell marker surfactant protein C (SPC) compared to control littermates (Fig. 4A, B, and Supplementary Fig. 5A for staining with secondary antibody only). The number of AECI cells was not affected as revealed by podoplanin immunofluorescence staining (Supplementary Fig. 5B, C). However, RT-qPCR data showed a significant reduction in podoplanin (*Pdpn*) expression in *Pkdcc*^−/−^ embryos (Supplementary Fig. 5D), possibly as a result of reduced expression of this gene in AECI or other cell types. The secretoglobin family 1A member 1 (*Scgb1a1*), a marker for Club cells [28], was expressed at similar levels in E18.5 lungs from CTRL and *Pkdcc*^−/−^ mice (Supplementary Fig. 5E). However, the area stained with an antibody against the bronchial epithelial cell marker cytokeratin 19 (CK19) [29] was significantly reduced in *Pkdcc*^−/−^ lung tissue (Fig. 4C, D). The bronchial wall area was also reduced, while the thickness of the bronchi and their total number was not significantly affected (Fig. 4E and Supplementary Fig. 5A for secondary antibody staining only). These results indicate abnormalities in the structure of bronchi, rather than a reduction of their numbers.

Overall, these data suggest that lung epithelial differentiation is delayed and/or altered in *Col1a2-Pkdcc*^*−/−*^ homozygous embryos. The SRY-box transcription factor 9 (SOX9) is involved in epithelial cell proliferation and differentiation across organs, and its expression is usually downregulated in the developing lung at E16.5 [30]. Consistent with a delay in lung development, we detected significantly more SOX9-positive cells in *Pkdcc*^−/−^ *vs.* control lung tissue at E18.5 (Fig. 4F, G and Supplementary Fig. 5F for secondary antibody staining only). Vascularization also plays a key role in lung organogenesis [21]. However, there was no difference in vessel size or vessel number as revealed by CD31 staining (Fig. 4H, I), but abnormalities in their positioning or functionality cannot be excluded. Because the HH signaling cascade plays an important role during lung development and in the crosstalk between mesenchymal and epithelial cells [28, 29] and because of the previously identified link between VLK and the HH pathway [4, 5], we investigated the expression of several HH targets at the mRNA level. Surprisingly, expression of *Gli1* and *Ptch1* was mildly, although significantly downregulated in lungs of *Pkdcc*^−/−^ mice (Fig. 4J), while expression of *Ccnd1* and *Smo* was not affected (Supplementary Fig. 5G). The reduced expression of *Gli* and *Ptch1* may point to a mild defect in lung branching because of the key role of HH signaling in this process [33, 34]. Consistent with this assumption, mRNA levels of Fgf10 and its receptor Fgfr2b, which are also key regulators of lung branching [35, 36] were also reduced in *Pkdcc*^−/−^ mice (Fig. 4J).

Overall, these results support a delayed or defective differentiation of the lung epithelium in mice lacking VLK in mesenchymal cells. This is likely to result in respiratory problems, providing a possible explanation for their early postnatal lethality.

### VLK regulates the abundance of ECM proteins in the developing lung

**Fig. 5:**
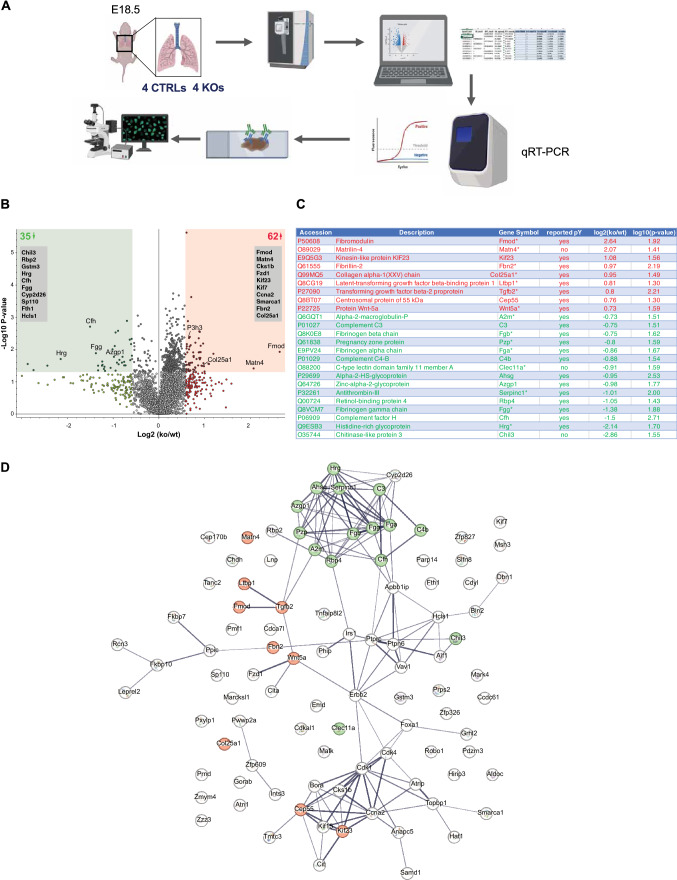
Quantitative proteomics analysis of E18.5 lung tissue reveals profound differences between CTRL and *Pkdcc*^−/−^ mice. **A** Workflow of proteomics analysis and validation, created with BioRender. **B** Volcano plot of all quantified proteins in lungs of CTRL vs.* Pkdcc*^*−/−*^ mice. Thresholds for selection of differentially abundant proteins: raw p value: 0.05, fold change: 1.5. Listed are the top ten proteins with either lower or higher abundance in samples from knockout *vs.* CTRL mice. **C** String database [37] (www.string-db.org) analysis of the 112 proteins fulfilling criteria for differential abundance between genotypes. Proteins classified as ‘GOCC:0005576 extracellular region’ by COMPARTMENTS [38] are highlighted and color-coded for significantly higher (red) or lower (green) abundance in samples from knockout *vs*. CTRL mice. **D** Table showing details for the 25 proteins highlighted in **C**). Asterisks indicate proteins that are also components of the mouse matrisome [39]. Information on reported tyrosine phosphorylation (pY) was extracted from PhosphoSitePlus^®^ v6.6.0.4 [51] (www.phosphosite.org)

To identify proteins, whose abundance is affected by the loss of VLK in mesenchymal cells and which may affect epithelial cells, we performed an unbiased mass spectrometry-based quantitative proteomics analysis of lung tissue from E18.5 *Pkdcc*^*−/−*^ mice and control littermates (Fig. 5A). In total, 6989 proteins were identified, of which 4627 could be relatively quantified through protein-level TMT labelling (Supplementary Table S1). Among them, 97 proteins were significantly and at least 1.5-fold differentially abundant (raw p value < 0.05) (62 increased, 35 decreased) between genotypes (Fig. 5B) (Supplementary Table S2). String database [37] (https://string-db.org/) analysis identified strong interactions between subsets of differentially abundant proteins and classified 24 of them as located in the extracellular region (GOCC:0005576) (Supplementary Table S3) as one of the top ten enriched COMPARTMENTS [38] categories. Fifteen of these proteins also belong to the mouse matrisome [39] (Fig. 5C, D), further pointing to alterations in the matrix of the knockout mice as suggested by the histological and biochemical data. Based on the increase in DHLNL cross-links that we observed (Fig. 3J), we checked if enzymes involved in collagen hydroxylation are differentially abundant. Among the detected enzymes we found that the abundance of multifunctional procollagen lysine hydroxylase was not altered, but prolyl 3-hydroxylase 3 (P3h3), which is part of a complex that also catalyzes lysine hydroxylation [40], was significantly increased in abundance in the knockout mice (Fig. 5B).

Analysis of 98 unique quantifiable tyrosine-phosphorylated peptides from the same dataset (Supplementary Table S4) identified the fibrinogen beta chain as significantly differentially lower in abundance in samples from *Pkdcc*^*−/−*^ vs. control littermates. The affected phosphorylation site (Y4) is located within the proteolytically released fibrinopeptide B. Notably, the effect size of this difference in abundance between genotypes (log2 = 0.592) was almost twice as high as for the corresponding fibrinogen beta protein (0.33), indicating a *bona fide* differential phosphorylation event.

**Fig. 6:**
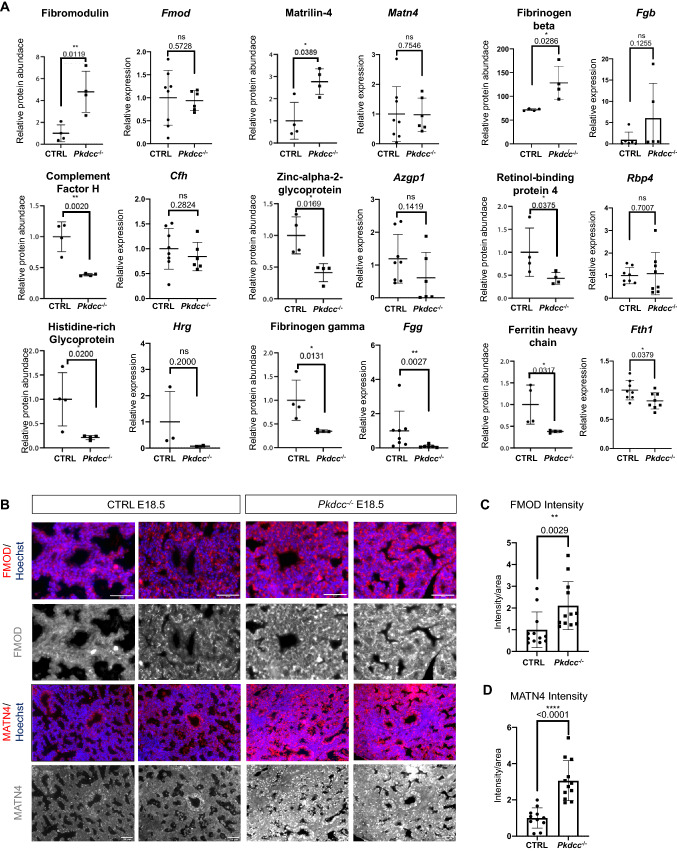
Verification of differential abundance of fibromodulin and matrilin-4 in E18.5 lungs of CTRL *vs Pkdcc*^−/−^ mice. **A** Bar graphs showing relative abundance of individual proteins based on the MS data and corresponding mRNA levels determined by RT-qPCR analysis (relative to *Rps29*). N = 4 mice per genotype (MS data) or 2–8 mice per genotype (RT-qPCR). **B** Representative immunofluorescence stainings of E18.5 lung sections from CTRL and *Pkdcc*^−/−^ mice for FMOD or MATN4 (red or white) and counterstaining with Hoechst (blue). Scale bars: 50 μm. **C**, **D** Quantification of FMOD (**C**) or MATN4 (**D**) staining intensities. N = 11–12 mice per genotype. Bar graphs show mean ± S.D. P values are indicated in the graphs; statistical significance was determined using Mann–Whitney U test (C, D and RT-qPCR in A). For "Relative protein abundance", p-values were taken from the MS analysis as described in Fig. 5B

The differential abundance of other quantified proteins suggests that alterations in a few specific non-collagen ECM proteins might drive the reported lung phenotype in *Col1a2-Pkdcc*^*−/−*^ embryos. In line with VLK’s reported function as a secreted kinase [1], we checked if these and other differentially abundant proteins are regulated at the protein rather than the RNA level. Interestingly, fibromodulin (FMOD), matrilin-4 (MATN4), fibrinogen beta (FGB), complement factor H (CFH), zink-alpha-2-glycoprotein (AZGP1) and retinol-binding protein 4 (RBP4) fulfilled this criterium (Fig. 6A), while others, including histidine-rich glycoprotein (HRG), fibrinogen gamma (FGG) and ferritin heavy chain (FTH1), also showed alterations in their mRNA levels (Fig. 6A). FMOD and MATN4 protein levels were approximately 6 or 4 times, respectively, higher in lung tissue from *Col1a2-Pkdcc*^*−/−*^ embryos (Figs. 5D and 6A).

To validate the proteomics findings, we determined FMOD and MATN4 expression in lung tissue sections from E18.5 embryos by immunofluorescence staining and confirmed the increase in FMOD and MATN4 in the lung of *Col1a2-Pkdcc*^*−/−*^ embryos (Fig. 6B-D and Supplementary Fig. 6 for secondary antibody staining only). MATN4 belongs to the matrilin family, which are matrix proteins that mainly serve as adaptors to link different ECM proteins, thereby building a supramolecular structure [33, 34]. FMOD is a small leucine-rich proteoglycan that is involved in collagen fibrillogenesis [43]. The differential abundance of these proteins therefore suggests that loss of mesenchymal VLK causes alterations in matrix organization and structure.

## Discussion

In this study we provide evidence for a key role of mesenchyme-derived VLK in lung development via its role in the regulation of the lung ECM and resulting alterations of lung epithelial cell differentiation. Knockout of *Pkdc*c in mesenchymal cells caused early postnatal death of the mutant mice. Surprisingly, very few *Pkdcc*^−/−^ mice, in particular male mice, survived to adulthood and did not exhibit major abnormalities, possibly as a result of unknown compensatory mechanisms, which may be more active in males. Alternatively, the Cre-mediated deletion may have been less efficient in these mice, resulting in compensatory growth of cells, which had escaped recombination. Such a phenomenon is frequent in mice with deletion of an essential gene [37, 38].

We propose that delayed or incomplete lung organogenesis likely results in impaired gas exchange and is the major cause for the very early postnatal lethality. In addition, mild alterations in the nasal bone and airway passage may further aggravate any breathing problems.

The reduction in AECII in *Col1a2-Pkdcc*^*−/−*^ lungs most likely results in insufficient surfactant production, leading to reduced oxygenation in the neonatal mice. Similarly, human premature infants have breathing difficulties due to the incomplete differentiation of surfactant-producing AECII cells and need surfactant supplementation to reduce tension within their lungs [44].

In line with an overall impaired lung organogenesis, the bronchial structures were also altered as reflected by reduced CK19 staining and the smaller bronchial wall area. Finally, expression of the progenitor marker SOX9, which during lung development is normally downregulated by E16.5 [30], was still strongly expressed in E18.5 *Col1a2-Pkdcc*^*−/−*^ tissue. This data further supports a delay or arrest in lung epithelial cell differentiation. It is possible that lung branching is also mildly affected in the mutant mice as suggested by the reduced expression of HH targets and of the genes encoding FGF10 and its receptor FGFR2, which are crucial regulators of this process [33–36]. Therefore, this possibility should be tested in future studies using whole-mount immunostaining.

Although some deletion in other cell types cannot be fully excluded, the results presented here strongly suggest non-cell-autonomous functions of VLK in the developing lung, which may be mediated by alterations of the matrix. However, we neither observed enhanced mesenchymal cell proliferation nor alterations in the extent of apoptosis in the lung of *Col1a2-Pkdcc*^*−/−*^ mice at E18.5, suggesting that the histological alterations are not a consequence of a higher number of mesenchymal cells. There was also no increased expression of collagens I and III, and the amount of total collagen was also unchanged. By contrast, DHLNL collagen cross-links were increased in E18.5 *Col1a2-Pkdcc*^*−/−*^ lungs, suggesting potential differences in tissue stiffness.

Quantitative proteomics comparing lung tissue from E18.5 *Col1a2-Pkdcc*^*−/−*^ mice and control littermates identified a significantly increased abundance of FMOD and MATN4 in *Col1a2-Pkdcc*^*−/−*^ lungs. FMOD forms a complex with and guides lysyl oxidase to specific collagen cross-linking sites [47], and the increased FMOD levels may therefore contribute to the observed differences in DHLNL cross-links. Similarly, the increase in MATN4 could also lead to a denser and potentially stiffer ECM due to its function in linking ECM components [33, 34].

The increased FMOD and MATN4 levels in the lung of *Col1a2-Pkdcc*^*−/−*^ mice were not associated with increased levels of their mRNAs, suggesting that their abundance is regulated at the protein level. Further studies should address whether VLK directly phosphorylates these proteins, thereby affecting for example their stability. The FMOD protein has a tyrosine residue (Y319), which had been shown to be phosphorylated (phosphosite.org), and VLK may be responsible for its phosphorylation. Members of the matrilin family have several conserved tyrosines, which up to now have not been reported as being phosphorylated. However, the strong conservation of some of these residues points to important biological functions, and it may well be that these tyrosines get phosphorylated under certain conditions.

Although inherently very limited due to lack of a specific enrichment step, a search for tyrosine phosphorylated peptides revealed differential phosphorylation of Y24 in the fibrinogen beta chain that corresponds to Y4 of the proteolytically released fibrinopeptide B. These data are not sufficient to validate fibrinogen beta as a new and direct VLK substrate, but are consistent with reports on fibrinogen tyrosine phosphorylation in the context of VLK activity [42, 43]. The reduced Tyr-phosphorylation of fibrinopeptide B in the *Pkdcc*^−/−^ mice may provide an explanation for the overall reduction in total fibrinogen beta, possibly because of protein destabilization. This may further contribute to the alterations in the ECM. Tyrosine phosphorylated peptides in MATN4 and FMOD were not detected in this study. Therefore, their potential phosphorylation should be tested in the future using a phosphopeptide enrichment approach.

Overall, our study shows that mesenchyme-derived VLK is required for proper lung organogenesis, an activity that results at least in part from non-cell-autonomous effects on lung epithelial cells. VLK is a secreted kinase, which phosphorylates substrates (including ECM and secreted proteins) in the secretory pathway and extracellularly [1]. Therefore, VLK is not only localized in or around the producing cells, but also in their microenvironment, and it seems likely that VLK produced by mesenchymal cells affects the neighboring epithelial cells. In addition, the phosphorylated substrates of VLK can affect the cells that produce VLK, but also other cell types in the close environment. A paracrine mechanism of action of VLK was previously also demonstrated in the liver, where the loss of VLK in hepatocytes promoted perivascular fibrosis, a phenotype that resulted from altered matrix production by mesenchymal cells [8].

Importantly, alterations in ECM deposition are a common feature of several lung pathologies in humans [49]. Therefore, it will be interesting in the future to determine the expression and function of VLK in developmental and fibroproliferative diseases of this organ.

## Supplementary Information

Below is the link to the electronic supplementary material.6989 proteins could be identified, from which 4627 could be relatively quantified through protein-level TMT labeling.

Supplementary file1 (XLSX 1305 KB)

Supplementary file2 (PPTX 41908 KB)

Supplementary file3 (DOCX 14 KB)

## Data Availability

The discovery mass spectrometry proteomics data have been deposited to the ProteomeXchange Consortium (http://proteomecentral.proteomexchange.org) via the PRIDE [50] partner repository with the dataset identifier PXD037700 and 10.6019/PXD037700.
