# Supplementary material for: Mesenchyme-derived vertebrate lonesome kinase controls lung organogenesis by altering the matrisome
Source: Cell Mol Life Sci. 2023 Mar 15;80(4):89. doi: 10.1007/s00018-023-04735-6 (PMC10017657; doi:10.1007/s00018-023-04735-6)
Supplement: Supplementary file 2 — Supplementary file2 (PPTX 41908 KB) [file 18_2023_4735_MOESM2_ESM.pptx]

## Slide 1
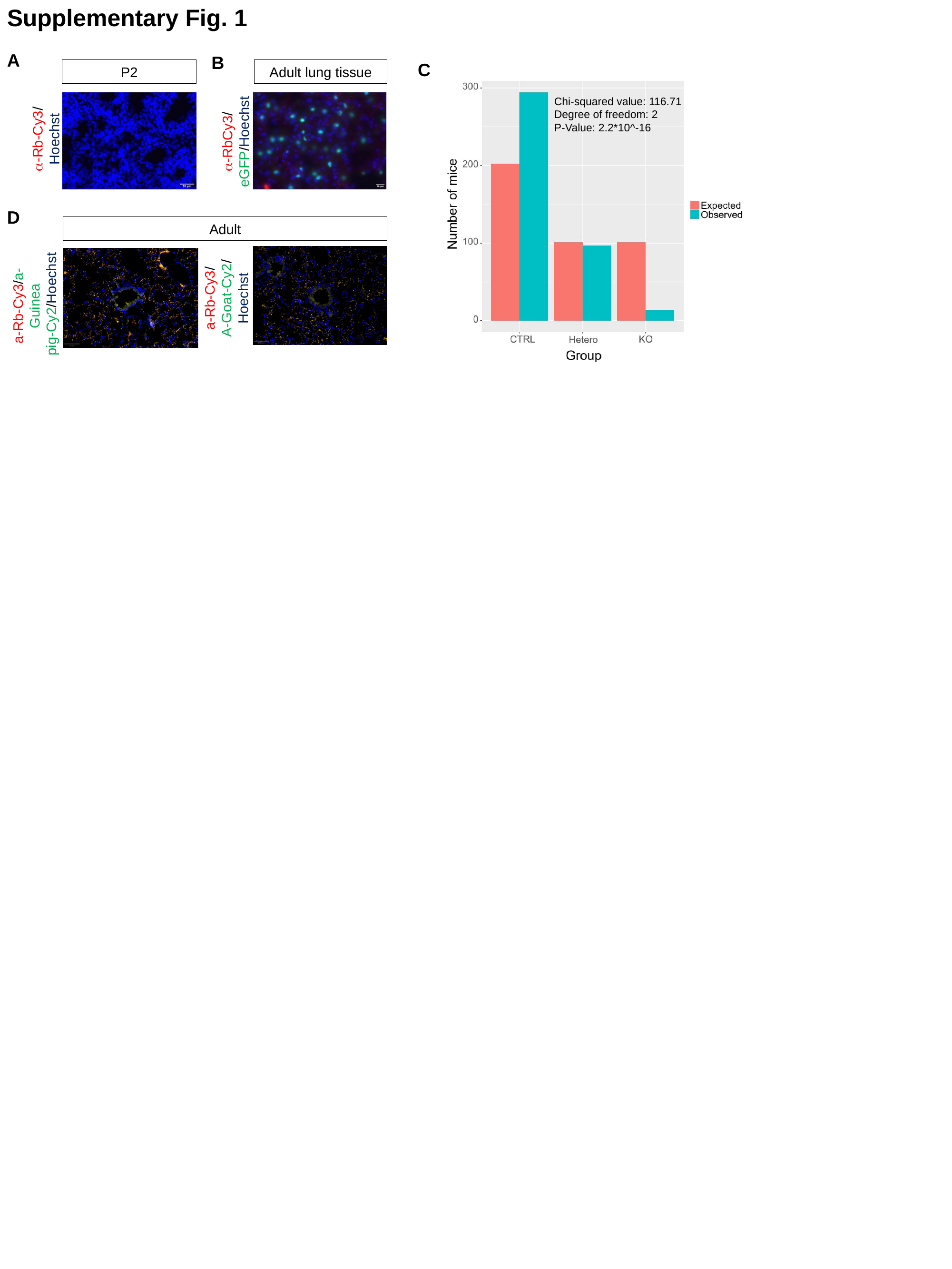

Supplementary Fig. 1
A
B
C
P2
a-Rb-Cy3/ Hoechst
Adult lung tissue
Chi-squared value: 116.71
Degree of freedom: 2
P-Value: 2.2*10^-16
a-RbCy3/
eGFP/Hoechst
D
Adult
a-Rb-Cy3/
A-Goat-Cy2/
Hoechst
a-Rb-Cy3/a-Guinea
 pig-Cy2/Hoechst

## Slide 2
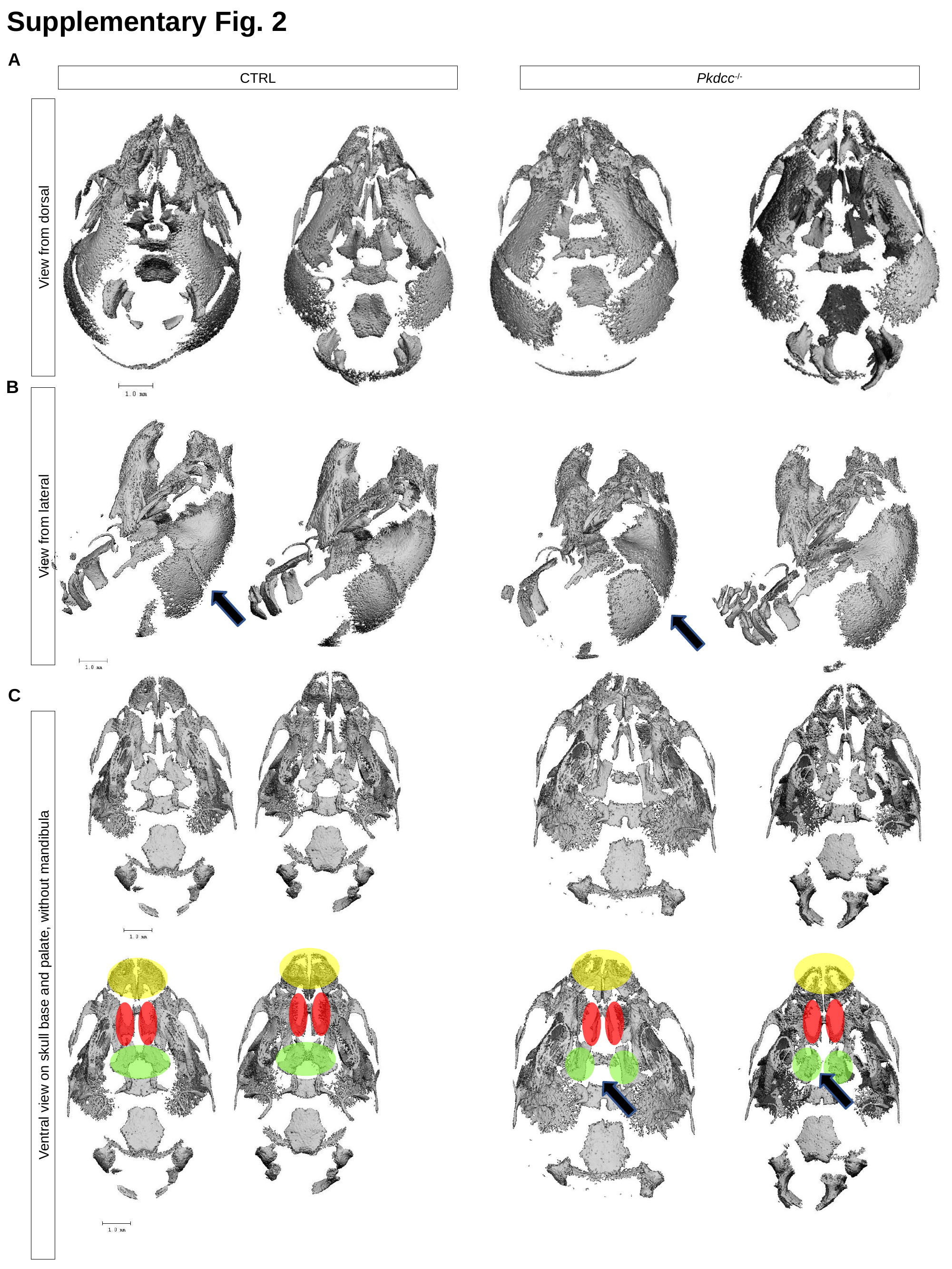

Supplementary Fig. 2
A
CTRL
Pkdcc-/-
View from dorsal
B
View from lateral
C
Ventral view on skull base and palate, without mandibula

## Slide 3
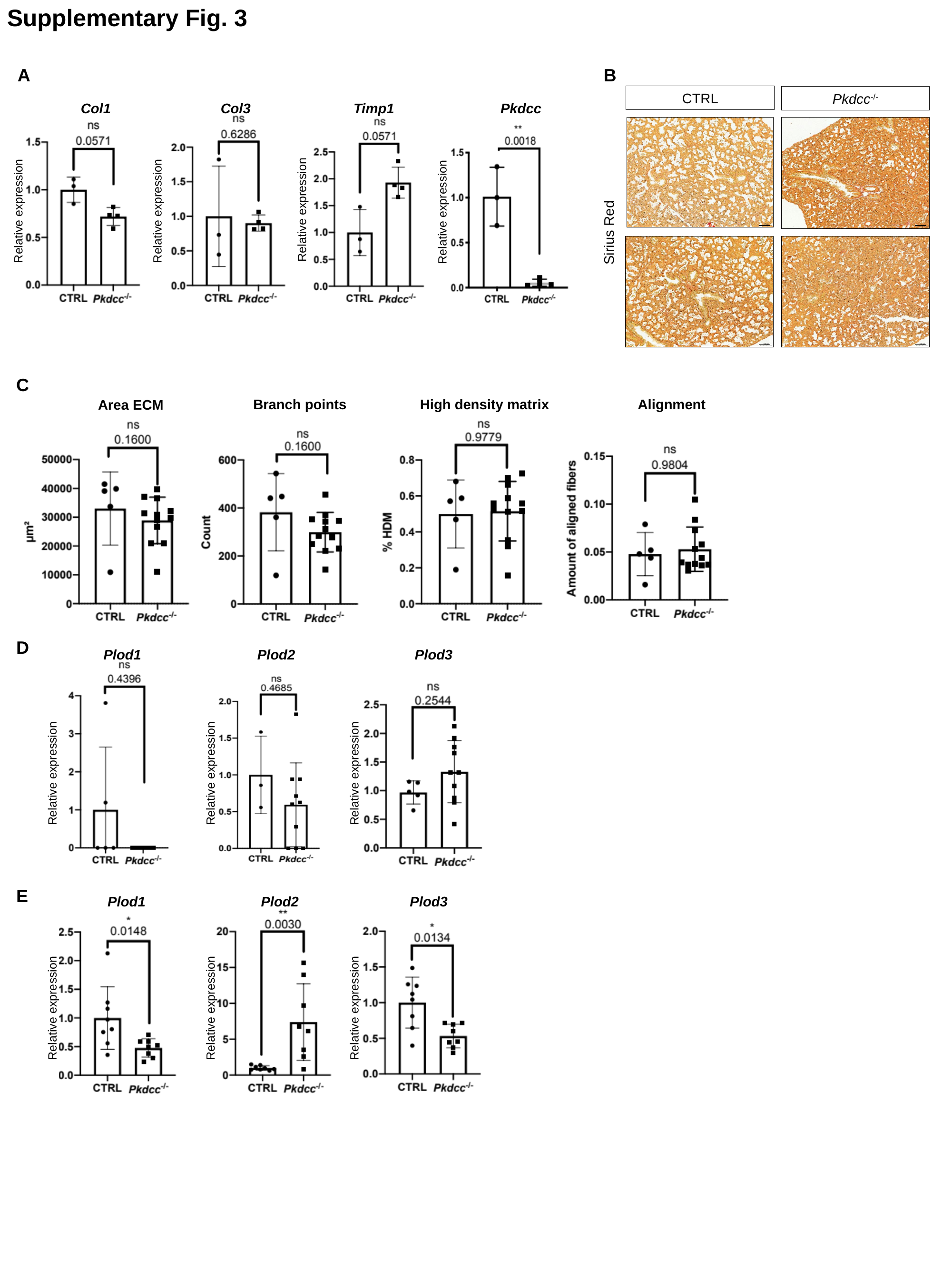

Supplementary Fig. 3
B
A
Col1
Pkdcc
Col3
Timp1
Relative expression
Relative expression
Relative expression
Relative expression
CTRL
Pkdcc-/-
Sirius Red
C
Alignment
High density matrix
Branch points
Area ECM
D
Plod3
Plod2
Plod1
Relative expression
Relative expression
Relative expression
E
Plod1
Plod2
Plod3
Relative expression
Relative expression
Relative expression

## Slide 4
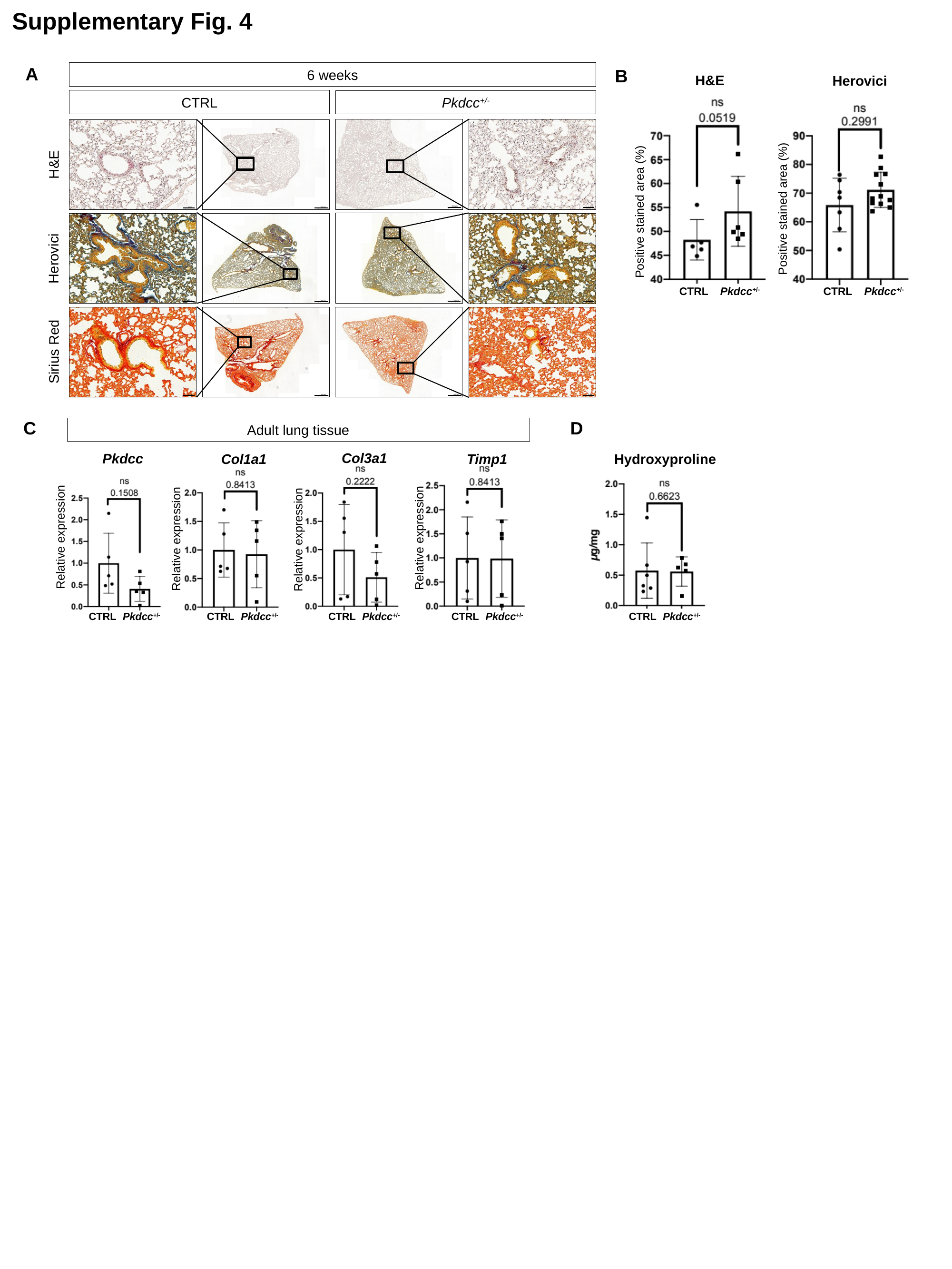

Supplementary Fig. 4
A
B
6 weeks
H&E
Herovici
CTRL
Pkdcc+/-
H&E
Positive stained area (%)
Positive stained area (%)
Herovici
CTRL
Pkdcc+/-
CTRL
Pkdcc+/-
Sirius Red
C
D
Adult lung tissue
Col3a1
Pkdcc
Col1a1
Hydroxyproline
Timp1
Relative expression
Relative expression
Relative expression
Relative expression
CTRL
Pkdcc+/-
CTRL
Pkdcc+/-
CTRL
Pkdcc+/-
CTRL
Pkdcc+/-
CTRL
Pkdcc+/-

## Slide 5
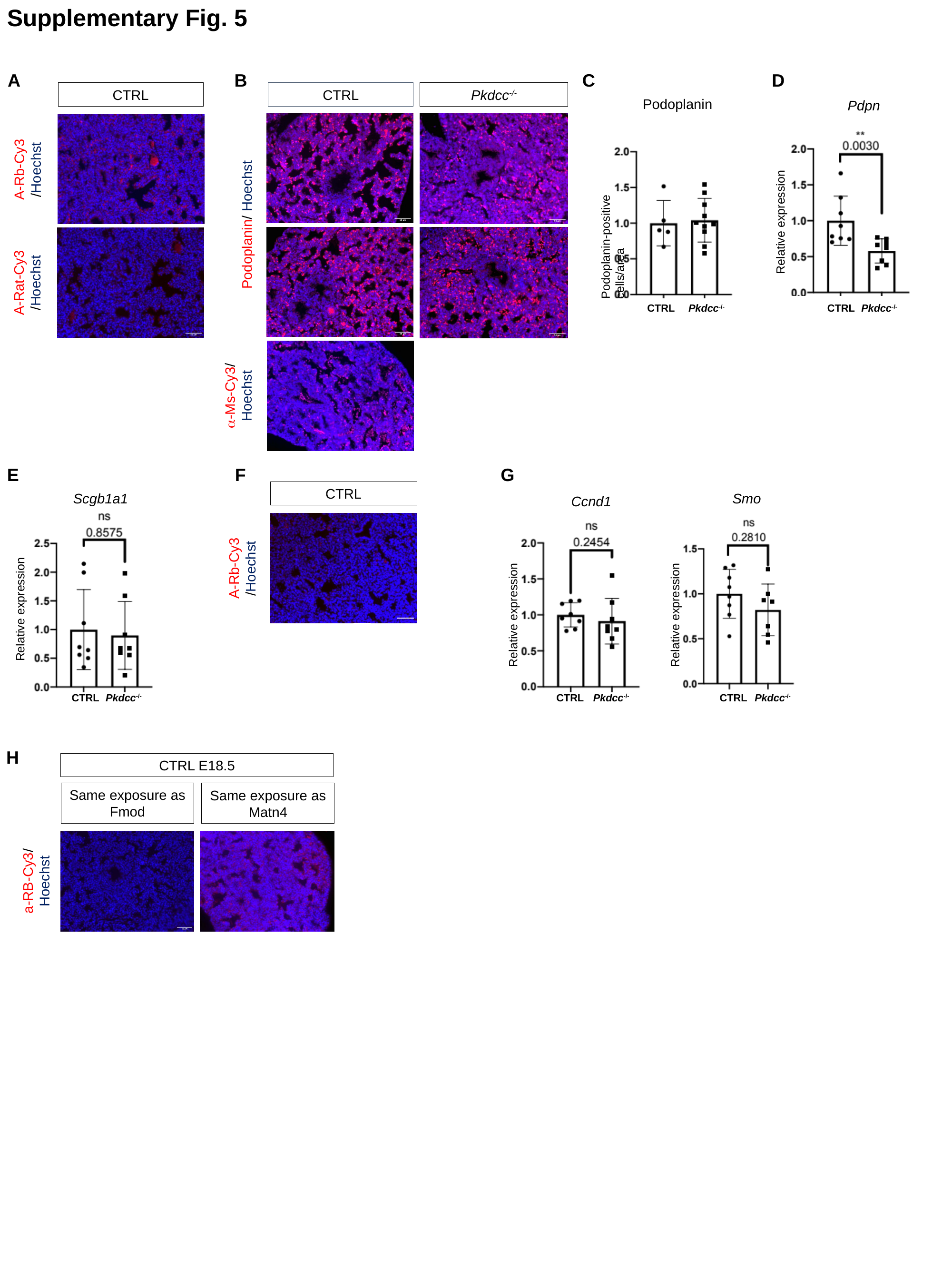

Supplementary Fig. 5
B
D
Pdpn
Relative expression
A
C
Podoplanin
CTRL
A-Rb-Cy3
/Hoechst
A-Rat-Cy3
/Hoechst
CTRL
Pkdcc-/-
Podoplanin/ Hoechst
a-Ms-Cy3/Hoechst
Podoplanin-positive cells/area
CTRL
Pkdcc-/-
CTRL
Pkdcc-/-
E
F
G
CTRL
A-Rb-Cy3
/Hoechst
Smo
Scgb1a1
Ccnd1
Relative expression
Relative expression
Relative expression
CTRL
Pkdcc-/-
CTRL
Pkdcc-/-
CTRL
Pkdcc-/-
H
CTRL E18.5
Same exposure as Fmod
Same exposure as Matn4
a-RB-Cy3/Hoechst

## Slide 6
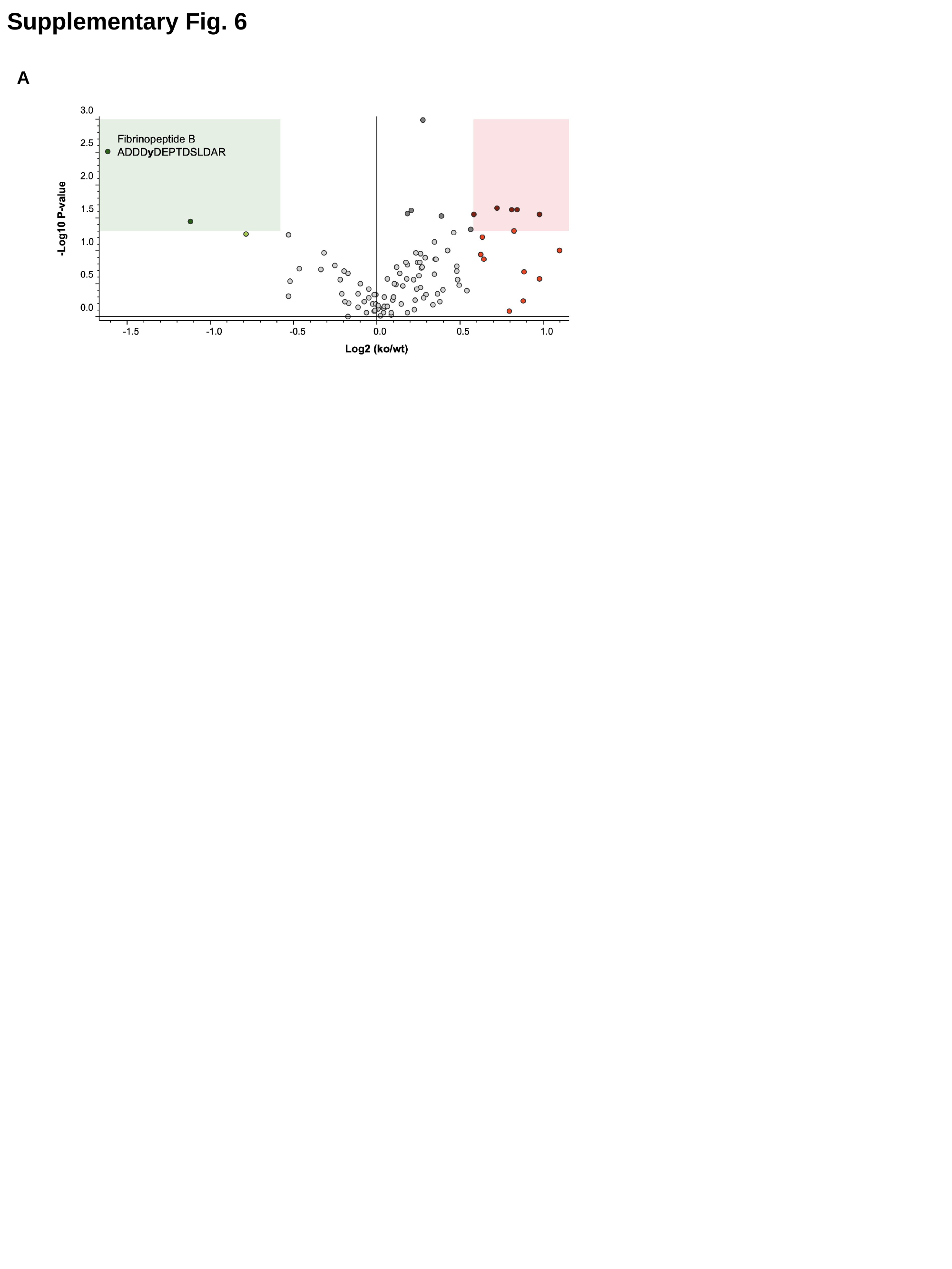

Supplementary Fig. 6
A
