# Supplementary material for: Mesenchyme-derived vertebrate lonesome kinase controls lung organogenesis by altering the matrisome
Source: Cell Mol Life Sci. 2023 Mar 15;80(4):89. doi: 10.1007/s00018-023-04735-6 (PMC10017657; doi:10.1007/s00018-023-04735-6)
Supplement: Supplementary file 3 — Supplementary file3 (DOCX 14 KB) [file 18_2023_4735_MOESM3_ESM.docx]

**Supplementary Fig. 1: Negative controls for VLK immunofluorescence staining**

**A, B, D)** Representative stainings of lung sections from P2 CTRL (A), adult Pdgfra-eGFP (B), or adult CTRL mice (D) using the secondary antibody only. Scale bars: 50 μm (P2), 20 μm (adult), or 50 μm (adult).

**C)** Chi-square survival test revealing a significant difference between the observed and expected Mendelian ratio of *Pkdcc*^-/-^ and CTRL mice.

**Supplementary Fig. 2: Craniofacial abnormalities in E18.5 *Pkdcc* knockout embryos**

**A-C)** CT Scans of the heads of E18.5 embryos showing the heads from the dorsal (A), lateral (B) and ventral position (C). Left: two CTRL animals, right: two *Pkdcc*^-/-^ mice.

**A)** Dorsal view of the skull. Note the shortening of the facial bones.

**B)** Lateral view of the skull. The arrow points to the top of the calvaria, which is more rounded in the *Pkdcc*^-/-^ compared to CTRL mice. The shortening of the facial bone is also visible.

**C)** Ventral view of the skull base and palate. The mandibula was removed from the image.

Incisive bones are highlighted in yellow, the palatine process of the maxilla is marked in red and the palatine bones in green. Arrows point to the potential palate cleft.

**Supplementary Fig. 3: *Pkdcc* deletion in mesenchymal cells does not induce collagen-associated fibrosis**

**A)** RT-qPCR analysis of RNA samples from primary MEFs established from E18.5 CTRL and *Pkdcc*^-/-^ mice for *Col1a1*, *Col3a1* and *Timp1* relative to *Rps29.* N=3-4 MEF cultures, each culture is from a different mouse. Confirmation of the *Pkdcc*^-/-^ in these cells is shown in Fig. 2E.

**B)** Representative photomicrographs of Sirius Red-stained lung sections from E18.5 CTRL and *Pkdcc^-^*^/-^ mice. Scale bars: 100 μm.

**C)** Quantification of Sirius Red staining of CTRL and *Pkdcc^-^*^/-^ mice using QuPath showing total ECM area, count of branch points, high density matrix in % and amount of aligned fibers.

**D)** RT-qPCR analysis of RNA samples from primary lung fibroblasts established from E18.5 CTRL and *Pkdcc*^-/-^ mice for *Plod1*, *Plod2* and *Plod3* relative to *Rps29*. N=5-10 lung fibroblast cultures, each culture is from a different mouse. Confirmation of the *Pkdcc* knockout in these cells is shown in Fig. 2F.

**E)** RT-qPCR analysis of RNA samples from total lung tissue of E18.5 CTRL and *Pkdcc*^-/-^ mice for *Plod1*, *Plod2*, and *Plod3* relative to *Rps29*. N=8 mice per genotype. Confirmation of the *Pkdcc* knockout in these lungs is shown in Fig. 3H.

Bar graphs show mean +/- S.D. P-values are indicated in the graphs; statistical analysis was performed using Mann-Whitney U test.

**Supplementary Fig. 4: Lung histology is not obviously affected in adult *Pkdcc*^+/-^ mice. A, B)** Representative images (A) and quantification (B) of the indicated histological stainings performed on lung sections from adult control and heterozygous *Pkdcc*^+/-^ mice; N= 5-7 control and 6-12 heterozygous mice. Scale bars: 500 μm and 50 μm. **C)** RT-qPCR analysis of RNA samples from adult lung tissue of CTRL and *Pkdcc*^+/-^ mice for *Pkdcc*, *Col1a1*, *Col3a1* and *Timp1* relative to *Rps29*. N=5 mice per genotype. **D)** Hydroxyproline content in the lungs of adult CTRL and *Pkdcc*^+/-^ mice. N=5-6 mice per genotype. Bar graphs show mean +/- S.D. P-values are indicated in the graphs; statistical analysis was performed using Mann-Whitney U test.

**Supplementary Fig. 5: *Pkdcc* deletion in mesenchymal cells severely affects lung epithelial cells A)** Negative control staining for SPC and CK19 (see Fig. 4) using the secondary antibodies only. **B, C)** Representative immunofluorescence stainings of E18.5 lung sections from CTRL and *Pkdcc*^-/-^ mice for podoplanin (red,) counterstained with Hoechst (blue) (B) and quantification of podoplanin-positive alveolar type I cells (C). Staining with the secondary antibody only is shown below the podoplanin stainings. N=5-10 mice per genotype. **D, E)** RT-qPCR analysis of RNA samples from E18.5 lungs of control and *Pkdcc*^-/-^ mice for *Pdpn* (D) and *Scgb1a1* (E) relative to *Rps29*. N=8 per genotype. **F)** Negative control staining for SOX9 (see Fig. 4) using the secondary antibody only. **G)** RT-qPCR analysis of RNA samples from E18.5 lungs of CTRL and *Pkdcc*^-/-^ mice for *Ccnd1* and *Smo*, relative to *Rps29*. N=8 per genotype. **H)** Negative control stainings for FMOD and MATN4 (see Fig. 6) using the secondary antibody only. Bar graphs show mean +/- S.D. P-values are indicated in the graphs; statistical analysis was performed using Mann-Whitney U test. Scale bars in A, B and F: 50 µm. Each image depicts a single mouse.

**Supplementary Fig. 6: Volcano plot of all quantified phosphotyrosine peptides in E18.5 lungs of CTRL *vs.* *Pkdcc^-/-^* mice.** Thresholds for selection of differentially abundant peptides: raw p-value: 0.05, fold change: 1.5. Shown is the peptide sequence of fibrinopeptide B with tyrosine phosphorylation site and lower abundance in samples from *Pkdcc*^-/-^ *vs.* CTRL mice.
